# Midgut Microbiota of the Malaria Mosquito Vector *Anopheles gambiae* and Interactions with *Plasmodium falciparum* Infection

**DOI:** 10.1371/journal.ppat.1002742

**Published:** 2012-05-31

**Authors:** Anne Boissière, Majoline T. Tchioffo, Dipankar Bachar, Luc Abate, Alexandra Marie, Sandrine E. Nsango, Hamid R. Shahbazkia, Parfait H. Awono-Ambene, Elena A. Levashina, Richard Christen, Isabelle Morlais

**Affiliations:** 1 UMR MIVEGEC (IRD 224- CNRS 5290- UM1- UM2), Montpellier, France; 2 Laboratoire de Recherche sur le Paludisme, IRD-OCEAC, BP288, Yaoundé, Cameroun; 3 UMR 7138 Systématique Adaptation Evolution, Université de Nice-Sophia Antipolis, Parc Valrose, France; 4 CNRS UPR 9022, Inserm U963, Université de Strasbourg, Strasbourg, France; 5 Área Departamental de Engenharia Electrónica e Computação, Faculdade de Ciências e Tecnologia, Universidade do Algarve, Faro, Portugal; Institut Pasteur, France

## Abstract

The susceptibility of *Anopheles* mosquitoes to *Plasmodium* infections relies on complex interactions between the insect vector and the malaria parasite. A number of studies have shown that the mosquito innate immune responses play an important role in controlling the malaria infection and that the strength of parasite clearance is under genetic control, but little is known about the influence of environmental factors on the transmission success. We present here evidence that the composition of the vector gut microbiota is one of the major components that determine the outcome of mosquito infections. *A. gambiae* mosquitoes collected in natural breeding sites from Cameroon were experimentally challenged with a wild *P. falciparum* isolate, and their gut bacterial content was submitted for pyrosequencing analysis. The meta-taxogenomic approach revealed a broader richness of the midgut bacterial flora than previously described. Unexpectedly, the majority of bacterial species were found in only a small proportion of mosquitoes, and only 20 genera were shared by 80% of individuals. We show that observed differences in gut bacterial flora of adult mosquitoes is a result of breeding in distinct sites, suggesting that the native aquatic source where larvae were grown determines the composition of the midgut microbiota. Importantly, the abundance of *Enterobacteriaceae* in the mosquito midgut correlates significantly with the *Plasmodium* infection status. This striking relationship highlights the role of natural gut environment in parasite transmission. Deciphering microbe-pathogen interactions offers new perspectives to control disease transmission.

## Author Summary

During their development in the mosquito vector, *Plasmodium* parasites undergo complex developmental steps and incur severe bottlenecks. The largest parasite losses occur in the mosquito midgut where robust immune responses are activated. Variability in *P. falciparum* infection levels indicates that parasite transmission is the result of complex interactions between vectors and parasites, which rely on both genetic and environmental factors. However, in contrast to genetically encoded factors, the role of environmental factors in parasite transmission has received little attention. In this study, we characterized the midgut microbiota of mosquitoes derived from diverse breeding sites using pyrosequencing. We show that the composition of the midgut microbiota in adult mosquitoes exhibits great variability, which is likely determined by bacterial richness of the larval habitats. When field mosquitoes were collected at late immature stages in natural breeding sites and the emerging females challenged with *Plasmodium falciparum* in the laboratory, significant correlation was observed between *P. falciparum* infection and the presence of *Enterobacteriaceae* in the mosquito midgut. Greater understanding of these malaria-bacteria interactions may lead to novel malaria control strategies.

## Introduction

Understanding how *Plasmodium*-*Anopheles* interactions contribute to the mosquito vector competence has received great attention lately, and the increasing knowledge promises to contribute to the development of new malaria control strategies [1]–[3]. Malaria still remains a serious health problem in developing African countries, causing more than 1 million deaths annually. Almost all these deaths are caused by the parasite *Plasmodium falciparum* whose major vector in Africa is *Anopheles gambiae*, which is widely distributed throughout the afro-tropical belt. *A. gambiae s.s.* is divided into two morphologically indistinguishable molecular forms, known as M and S, which are regarded as incipient species [4]–[6]. The M and S molecular forms exhibit ecological preferences [7], [8], but their respective epidemiological importance in malaria transmission has been poorly documented so far [9], [10].

The susceptibility of *Anopheles* mosquitoes to *Plasmodium* infection is under genetic control [11]–[13], but the large variability in oocyst number among closely related mosquitoes indicates that environmental factors also play a role. Multiple lines of evidence suggest that mosquito bacterial communities influence vector competence [14]–[17]. A protective role of *Anopheles* midgut bacteria against malaria infections was demonstrated by using antibiotic treatment to clear the gut microbiota, which resulted in enhanced *Plasmodium* infections [15], [18]. Consistently, coinfections of bacteria with *Plasmodium* reduced the number of developing oocysts in the mosquito midgut, both in laboratory and field conditions [15], [19], [20]–[24]. Interestingly, Cirimotich *et al.* [24] recently described an *Enterobacter* bacterium isolated from wild mosquitoes in Zambia that confers refractoriness to *P. falciparum* infection. Mechanisms mediating this refractory phenotype remain elusive. Instead of eliciting immune responses leading to reduced levels of parasite burden, the experiments conducted by Cirimotich *et al.* [24] revealed that the inhibition of *Plasmodium* development by commensal microbiota occurs through production of reactive oxygen species by the *Enterobacter* bacteria that directly target *Plasmodium* parasites in the midgut [17], [24].

Bacterial diversity in the *Anopheles* species is thought to be particularly low at the adult stage because of gut renewal during metamorphosis from pupae to adults. Nevertheless several bacterial species have been identified in the adult mosquito midgut using different conventional culture-mediated techniques [16], [23], [24]. These bacteria were acquired from the aquatic environment during immature stage development [25], [26], although vertical transmission (from mother to offspring) also has been documented [14], [16], [25], [27]. Generally, knowledge on mosquito midgut bacterial communities remains largely unknown, mostly because of the limitations of isolating techniques based on culturing and to the low resolution of fingerprint analysis. However, the recent deployment of next generation DNA sequencing technologies has provided new opportunities to explore microbial diversity of complex environments [28]–[31] as well as to further investigate disease susceptibilities and host-bacteria-pathogen interactions [32]–[34].

In this study, we performed a meta-taxogenomic analysis of microbial communities in the midguts of adult mosquitoes originating from natural larval habitats in Cameroon. We further investigated correlations between midgut microbiota and the mosquito malaria infection status. Previous investigations of bacteria-*Plasmodium* interactions in the mosquito vector have considered laboratory-reared mosquitoes challenged with cultured bacteria and infected with a cultured *P. falciparum* line. Here, we challenged wild female mosquitoes with a natural isolate of *P. falciparum*, thereby offering an opportunity to examine natural bacteria/parasite associations. Our analysis revealed that the midgut bacterial diversity represents an important force shaping the mosquito vector competence, where bacteria of the *Enterobacteriaceae* genera benefit *P. falciparum* development.

## Results

### Mosquito susceptibility to *P. falciparum* and bacterial flora

A total of 92 *A. gambiae* mosquitoes collected at the larval stage and reared to adults in the insectary were successfully fed through membrane feeders on gametocyte containing blood from a single individual. The origin of mosquitoes and their genetic characteristics (molecular form and infection status) are summarized in Table S1. Mosquitoes of M molecular form were significantly more infected than those of S form (48.5% versus 27.1%; OR 0.39; 95% CI: 0.15–1.06; *P* = 0.044). However, the comparison of infection prevalence (number of infected mosquitoes) between the different localities revealed a “sampling site effect” (Fisher's exact test *P*<0.01).

Of those challenged with *P. falciparum* we then investigated the gut microbiota in mosquitoes originating from two different breeding sites. We used field mosquitoes from Mvan and Nkolondom and gut bacterial communities determined for 8 *P. falciparum*-PCR positive and 7 negative mosquitoes from each locality (Table S2).

### Composition of microbial communities in mosquito midguts

Pyrosequencing of 16S rDNA generated a total number of 663,798 sequence reads across the 3 hypervariable regions S1, S2, and S3 in the bacterial gene in 32 mosquitoes (Table 1). Few individuals failed to amplify the SSU regions (2 for S1, 3 for S2, and 5 for S3), which was not linked to DNA quality as at least one region was successful for all samples, making it likely that technical problems in the PCR were responsible. After tag extraction and filtering of low-quality sequence tags, we obtained 575,284 reads for the analysis, representing 86.7% of the 454 reads. The average number of tags for all SSU regions combined per sample was 6,827 (±811), read number per gut ranging from 3,305 to 10,169. About 99.0% of sequence reads were successfully assigned, with unique tags representing 25.4% of the average tag number over the three SSU regions.

**Table 1:** Summary of pyrosequencing tags for the 3 amplified SSU rDNA regions.

	SSU rDNA region		
	S1	S2	S3
Total 454-reads	229,540	215,046	219,192
Dereplicated tags	206,592	167,440	201,252
Average tags per sample (SD)	6,886 (+/−1,601)	6,201 (+/−1,589)	7,454 (+/−1,559)
Assigned tags (%)	99,8	99,9	97,5
Unique tags (%)	24,5	28,2	23,7
Phylum number	26	17	15
OTUs at the family rank	140	116	96

**Figure 1:**
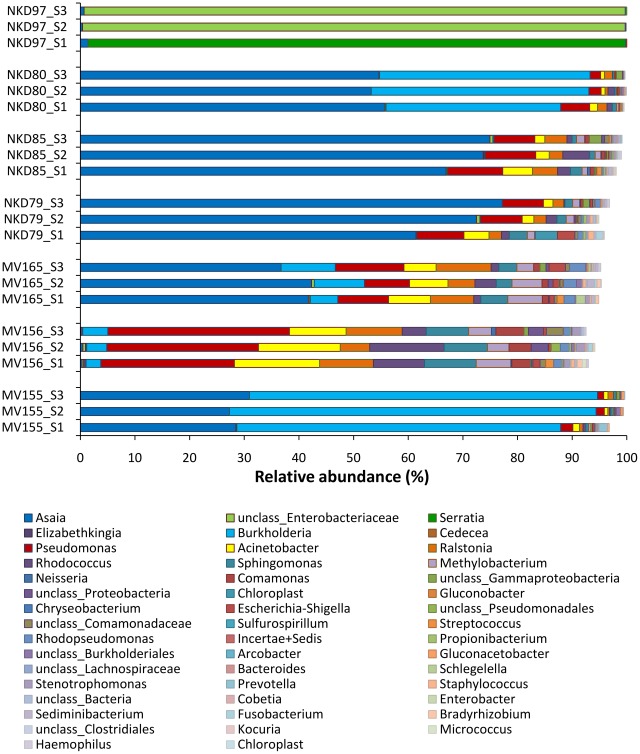
Comparison of bacterial diversity for the three 16S libraries at the genus level. Tag abundance was compared for three 16S libraries, and the graph shows the bacterial flora of six mosquito midguts. The three 16S libraries were obtained using primer sets targeting different 16S domains, as described in the Materials and Methods section. Only the most abundant categories (>2%) were considered. The S1 library only reached 95%, showing this domain allowed identifications for a greater number of minor clades. For mosquito NKD97, S2 and S3 primer sets only allowed the identification at the *Enterobacteriaceae* family level, whereas S1 reached the assignment at the genus level, *Serratia*.

We first compared the pyrosequencing data for the 23 gut samples that yielded sequence tags for all three 16S domains (Table 1). The comparison of the microbial communities between the 3 domains for seven midgut samples is given in Figure 1. The three 16S domains overall provided a very similar picture of the bacterial populations, even if they differed for the exact percentages. When only the most abundant taxonomic categories were considered (constituting >2% of the overall), the S1 domain reaches a lower percentage, indicating that this 16S library capable of identifying a greater number of minor clades (Figure 1). In addition, the S1 domain had better resolution, allowing more precise assigning of sequence tags (see Figure 1, mosquito NKD97). We then performed further analyses on the S1 domain, for all 30 samples that were successful for pyrosequencing.

**Figure 2:**
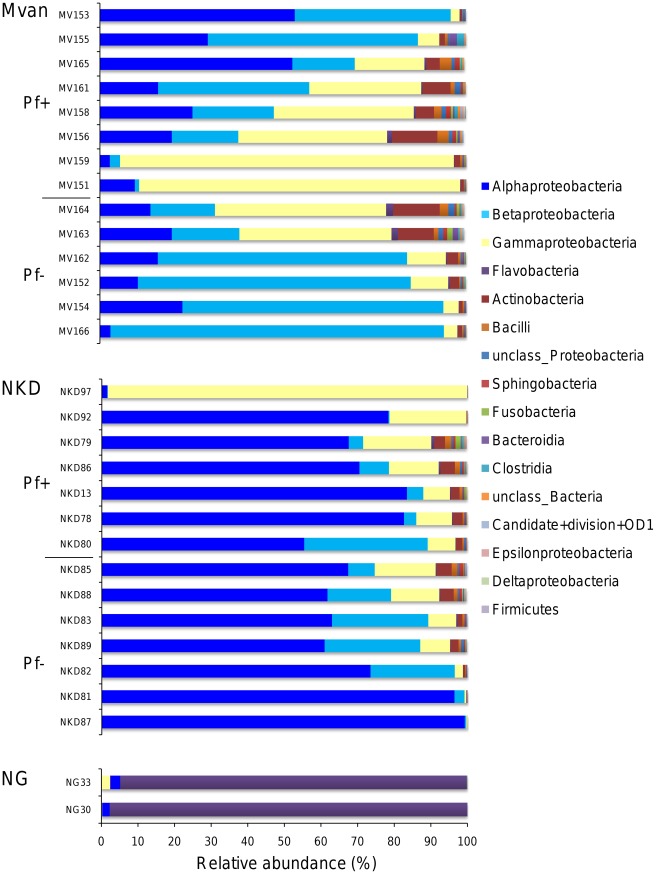
Relative abundance of the different bacterial classes within each mosquito midgut sample. Mvan and NKD (Nkolondon) indicate the geographical origin of mosquitoes, NG being mosquitoes of the laboratory colony Ngousso. Pf+ and Pf− designate the *P. falciparum* infection status of the challenged mosquitoes, positive and negative, respectively. Only class rank groups that represented >1% of the total reads, and identified in at least 30% of mosquitoes, are shown. “Unclass” represents tags that could not be assigned to the class level, and were grouped into a higher taxonomical rank.

The bacterial communities of the mosquito midgut belonged to 26 different phyla, among which, 5 represented more than 99% of the total microbiota: *Proteobacteria*, *Bacteroidetes*, *Actinobacteria*, *Firmicutes*, and *Fusobacteria*. We examined the relative abundance of the major classes, that is, detected in more than 30% of the samples and having an average abundance of >0.1% (Figure 2). The gut microbiota presented a large inter-individual variability and was dominated by few taxa. The first striking result came from laboratory-reared mosquitoes that exhibited a drastically different composition of midgut bacteria from field mosquitoes. More than 96% of tags corresponding to bacteria in the midguts of mosquitoes from the Ngousso colony were assigned to *Flavobacteria*, although this class accounted for only 0.38% (±0.24) of the sequence tags in field mosquitoes. Similarity searches against the SSU SILVA database (release 108) identified *Flavobacteria* tags as belonging to *Elizabethkingia sp.*, which already has been isolated from *A. gambiae* midguts from different insectaries [15], [35], [36]. The guts of Ngousso mosquitoes also contained, to a lower extent, *Gammaproteobacteria* (*Pseudomonas sp.*) and *Alphaproteobacteria* (*Asaia sp.*).

In mosquitoes from natural habitats, midguts were mainly colonized by *Proteobacteria* (94%), and the most prominent classes were *Gammaproteobacteria*, *Alphaproteobacteria* and *Betaproteobacteria* (Figure 2). Figure 1 clearly shows the difference in bacterial composition between mosquitoes from the 2 breeding sites used in this study. In mosquitoes originating from Nkolondom, the intestinal bacterial flora is dominated by *Alphaproteobacteria* (68.65±7.38%), mainly of the genus *Asaia sp*. By contrast, the three major classes are almost equally represented in the midgut of mosquitoes from Mvan, although with large individual variability. The main taxa in this locality were *Asaia*, *Sphingomonas*, *Burkholderia*, *Ralstonia*, and *Enterobacteriaceae*. *Enterobacteriaceae* could not be assigned to more precise taxonomic ranks.

Midgut bacteria were unevenly distributed among individual mosquitoes and between the different sampled localities. Several genera were found in all, or at least in a large majority, of the mosquitoes possibly representing the “mosquito midgut core microbiota” (Table S3). They included members of the genera *Asaia*, *Burkholderia*, *Serratia*, *Ralstonia*, *Acinetobacter*, *Pseudomonas*, *Sphingomonas*, *Staphylococcus*, *Streptococcus*, and *Escherichia/Shigella*. Of note, *Asaia sp.* was found in all samples, and its relative abundance showed great variation from one midgut to another, ranging from 1.49 to 98.95% in mosquitoes from Nkolondom and from 0.04 to 49.66% in those from Mvan. The group of unassigned *Enterobacteriaceae* also was identified in all field samples, with relative abundance ranging from 0.01 to 1.03% and from 0.04 to 71.51% in Nkolondom and Mvan, respectively. Other specific members of *Enterobacteriaceae* were frequent and represent a large proportion of the midgut bacterial communities: *Serratia spp.* accounted for 96.93% of the midgut bacteria in a mosquito (NKD97) from Nkolondom, and *Cedecea spp.* encompassed 12% of the gut bacterial content in 2 mosquitoes from Mvan. *Escherichia*/*Shigella* was found in more than 85% of the mosquitoes, at low densities. The sequence of the Esp_Z *Enterobacter* (JF690924), despite its presence in our reference database, was absent from the analyzed reads.

Of note, the midgut bacterial flora was mainly composed of Gram-negative communities. No Gram-positive bacterium was identified in the laboratory mosquitoes, whereas they represented 5% of the total microbiota of the field mosquitoes. Gram-positive bacteria belonged to the classes *Bacilli* and *Actinobacteria*.

To determine whether all phylotypes present in the mosquito midgut microbiota were detected in this study, we performed a rarefaction analysis for each sample on tags from the S1 domain; rarefaction curves are shown in Figure S1. The rarefaction curves decrease rapidly at approximately 2,000 sequences per sample and reach saturation at 3,000, indicating that our sequencing effort was sufficient to catch the overall bacterial diversity in the mosquito midgut. The rarefaction curves show the large variability in bacterial complexity among samples, varying from 13 to 340 operational taxonomic units (OTUs). In addition, they illustrate the paucity of clusters in the midgut of laboratory mosquitoes. They also revealed greater bacterial diversity in the samples from Mvan compared with those from Nkolondom (185±51 and 110±30, respectively; t-test *t* = 2.385, *P* = 0.025).

### Microbial diversity in the mosquito intestinal microbiota

The Chao1 estimator, which gauges the number of unseen “species,” predicted that we covered, on average, 81% of the species diversity across all samples. To confirm this result, we computed the ACE and Jackknife estimator indexes; both had higher values than the observed richness, indicating an underestimation of the gut microbial diversity (see Table S2).

We characterized the species diversity in our set of mosquito midguts using the species richness, the Shannon diversity index (*H*) and the Simpson's diversity index (*D*); data are shown in Table S2. No significant differences in the richness index were found comparing mosquito locality and/or *P. falciparum* prevalence. Significant differences of the diversity indexes were found when comparing mosquito locality (Shannon, *P* = 0.0091 and Simpson, *P* = 0.0097) but none when comparing the infection prevalence of mosquitoes. Thus, at the genus level, the microbiota of mosquitoes from Mvan was more diverse than that from Nkolondom, but *Plasmodium*-infected and non-infected mosquitoes did not exhibit differences in their microbial diversity.

**Figure 3:**
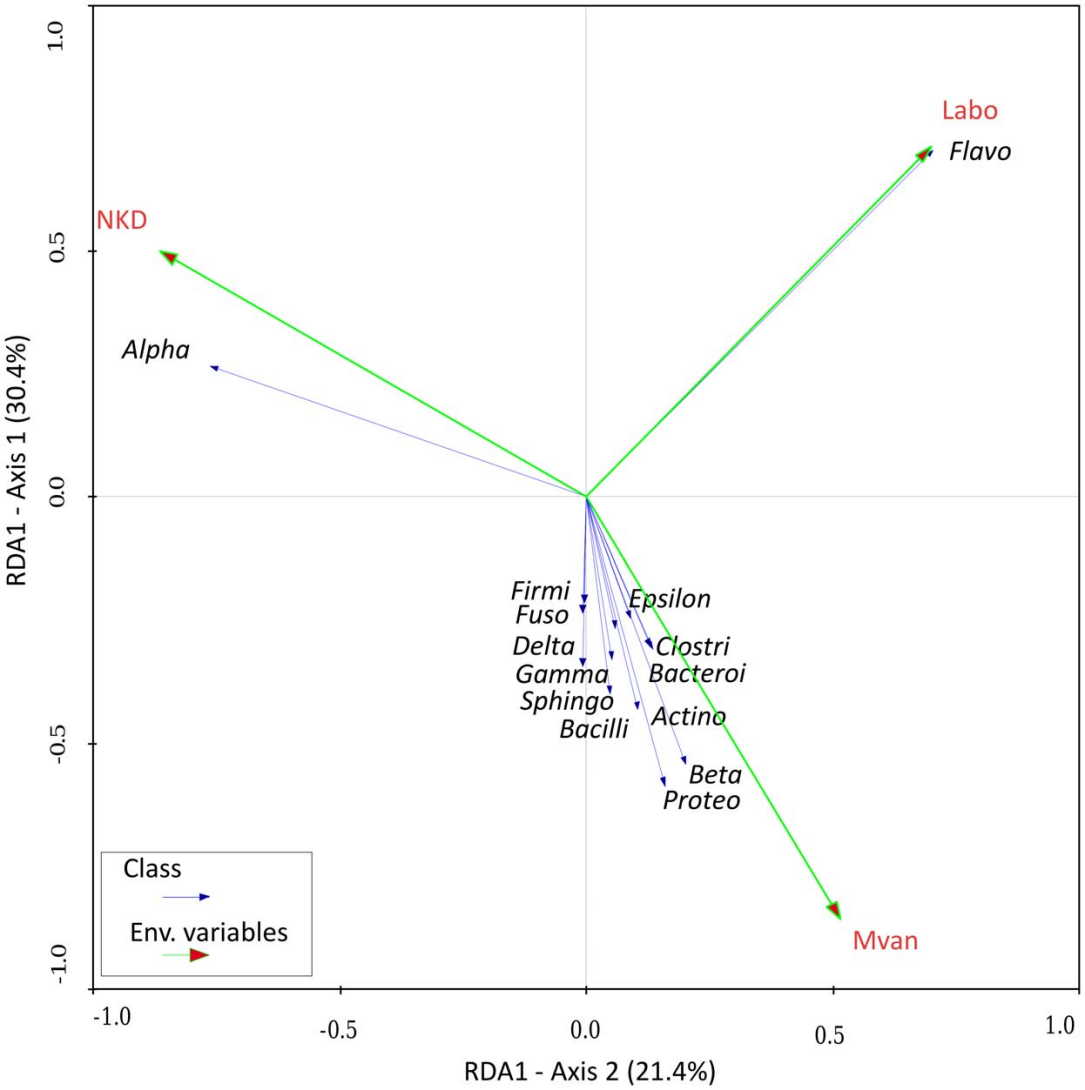
Redundancy analysis for gut bacterial communities (taxonomic rank = class) in field and laboratory mosquitoes. The length of arrows indicates the strength of correlation between the variable and the ordination scores. Blue arrow: bacterial classes, green arrow: environmental variables. The Monte Carlo permutation test was used to test the statistical significance of the relationship between environmental variables and the bacterial classes. The “Flavo” (*Flavobacteriaceae*) segregates with “labo” environmental variable, “Alpha” (*Alphabacteriaceae*) with the “NKD” environmental variable (*P*<0.05). All other bacterial classes segregate along the second axis, with the “Mvan” environmental variable.

The relationship between the class taxonomic rank of bacteria and the origin of the mosquitoes (locality) was evaluated using redundancy analyses (RDA) (Figure 3). The Eigen values of the first four axes were recorded at 0.304, 0.214, 0.331, and 0.144, respectively. The first two constrained axes explained around 50% of the total variance in the bacterial community and 100% of the species-environment relationship. The unrestricted Monte Carlo permutation test (n = 499) indicated that all environmental variables were significant (Nkolondom variable: *F* = 10.99, *P* = 0.002; Mvan: *F* = 13.26, *P* = 0.004; Mvan and laboratory variables fit collinearly). Thus the different classes of bacteria were not randomly distributed but linked to the breeding site where mosquitoes grew up. As seen in Figure 2, *Flavobacteria* were related to laboratory mosquitoes, whereas *Alphaproteobacteria* were less diverse and related to the Nkolondom locality. The remaining classes cluster along the Mvan locality. These results confirm the higher diversity of bacterial taxa in mosquitoes collected in Mvan as compared with Nkolondom, and the paucity of the gut microbiota in laboratory-reared mosquitoes as compared with mosquitoes from the wild.

### Relationship between gut microbial communities and *P. falciparum* prevalence

**Figure 4:**
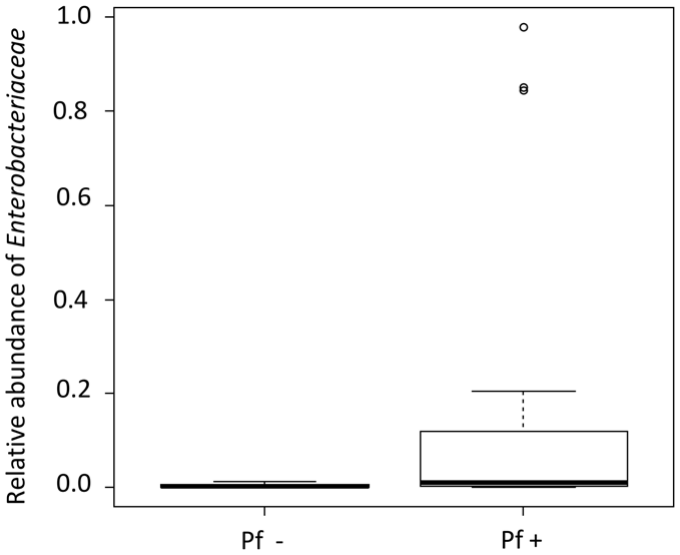
Relative abundance of *Enterobacteriaceae* in *P. falciparum* non-infected (Pf−) or *P. falciparum* infected (Pf+) mosquitoes. The *Enterobacteriaceae* loads are significantly higher in *P. falciparum* infected mosquitoes (*P* = 0.004; Mann–Whitney test). The boxes represent the interquartile range (25–75^th^ percentile), and the line within each box corresponds to the median value.

We then investigated potential relationships between the gut microbial communities of field mosquitoes and the *P. falciparum* infection status. We performed the RDA by plotting the infection status and the origin of the field mosquitoes against the family taxonomic rank, allowing the analysis of more precise bacterial taxa (Figure S2). The first and second constrained axes corresponded to 35% and 7% of the total variance in the bacterial community, respectively, and explained all the cumulative percentage variance of the family-environment relationship. All environmental variables were significant (Monte Carlo test, Nkolondom: *F* = 14.02, *P* = 0.002; collinearity detected with Mvan variables; infection variable *F* = 3.00, *P* = 0.042). The first axis alone explained 84.1% of the variance of the family environment relationship and was related to the mosquito origin (Mvan and Nkolondom). In concordance with the results already described for the *Alphaproteobacteria* class, the *Acetobacteriaceae* family was related to mosquitoes from Nkolondom, and most of the family is represented by *Asaia spp.* By contrast, the mosquitoes from Mvan exhibited a larger bacterial diversity. Interestingly, the RDA revealed a relationship between the *Enterobacteriaceae* family and the infection status along axis 2 (Figure S2). This result suggests that mosquitoes harboring *Enterobacteriacae* are more likely to be infected by *P. falciparum*. A correlation between the relative abundance of *Enterobacteriaceae* in the midgut and *P. falciparum* infection was further detected using the non-parametric Mann-Whitney test (*P* = 0.004; Figure 4), indicating that *P. falciparum*-positive mosquitoes were hosting more *Enterobacteriaceae* bacteria.

## Discussion

We provide here an in-depth description of the microbial communities in the midgut of the malaria mosquito. Using pyrosequencing, we explored individual midgut samples from adult female mosquitoes collected at the larval stage in different natural environments, exposed to *P. falciparum* infection at day 5 after emergence and dissected 8 days after the infectious blood meal. We then examined the microbial diversity according to the origin of the mosquitoes and investigated putative correlations between the bacterial content and the malaria infection status by comparing midgut microbiota in *P. falciparum*-positive and *P. falciparum*-negative individuals.

The adult mosquito midgut microbiota comprises five dominant phyla *Proteobacteria*, *Bacteroidetes*, *Actinobacteria*, *Fusobacteria*, and *Firmicutes* and presents some similarities with gut microbial communities from other invertebrate midguts, including mosquitoes [37]–[44]. Nonetheless, pyrosequencing revealed a higher diversity than more conventional molecular techniques, with an average of 147.64 OTUs (±88.49, at a 0.04% occurrence threshold) and an estimated richness of 72.27 (±31.70) taxa per field mosquito. Although bacterial richness is greater than previously described in mosquitoes, the vast majority of sequence tags (>90%) felt into few taxa and only 21 bacterial families, and 28 genera had an abundance of >1% in at least one mosquito midgut. Thus, the mosquito midgut is colonized by few dominant bacteria species, probably involved in metabolic functions.

We used three different pairs of primers (S1, S2, and S3) to amplify and analyze each midgut sample. The comparison of the bacterial diversity for the three libraries revealed similar patterns; however, the S1 library allowed more detailed identification of the mosquito microbiota. These data strengthen the previous observation that the SSU rRNA gene clone libraries are biased by the choice of the set of primers used for amplification and thereby distort the revealed biodiversity [45]. To our knowledge, this is the first 454 sequencing analysis where different couples of primers have been used to identify the bacterial diversity in biological samples. The analysis ensured that the primer sets used produced the most accurate view possible of the bacterial content of the mosquito midgut.

*Proteobacteria* represented more than 90% of the bacterial gut content in the mosquitoes from the wild, whereas in laboratory-reared mosquitoes, more than 95% of sequence tags belonged to the *Flavobacteria Elizabethkingia spp*. The remaining tags from laboratory mosquitoes were assigned to the members of *Gammaproteobacteria* (*Acinetobacter*, *Pseudomonas*), *Firmicutes* (*Staphylococcus*, *Streptococcus*), and the *Alphaproteobacterium Asaia* sp. Bacterial richness and diversity seem to be particularly poor in the laboratory mosquitoes. We identified the *Elizabethkingia spp.* in 68% (19/28) of the field-collected mosquitoes, at low densities (<0.5% of the total bacterial content), suggesting either that the bacterium has developed symbiotic associations with the mosquito midgut or that the bacterium is widespread in nature. The predominance of *Elizabethkingia spp.* in the midguts of the insectary-reared mosquitoes reflects that the bacterium has found a thriving niche in this environment where competition with other bacterial species is limited. *Bacteroidetes* are known as glucose degraders, and the large dominance of *Elizabethkingia spp.* in laboratory-reared mosquitoes is probably due to the mosquito food source [46]. Indeed, the midgut microbial diversity is directly associated with the individual diet [47]–[50]. In this study, all adult mosquitoes were maintained in our standard rearing conditions on a sterile glucose solution. The aquatic environment of the larval stages presented striking differences: larvae of the Ngousso strain were grown in clean spring water, whereas the immature stages of field mosquitoes were collected in natural breeding sites, water puddles, and flooded areas rich in biotic and abiotic components. Thus, our results indicate that the environmental conditions of the vectors are key determinants in shaping midgut microbiota. The drastic loss of microbial diversity from the wild to laboratory conditions may have important consequences on mosquito fitness and on the gut immune system. This undoubtedly explains the higher prevalence and intensity of *P. falciparum* infections in laboratory colonies of *A. gambiae* as compared with field-derived mosquitoes and pinpoints the limitations of using laboratory models to study host-pathogens interactions (Morlais and Cohuet, unpublished).

The great difference in the composition of gut bacteria between laboratory and field-collected mosquitoes as well as between mosquitoes originating from distinct breeding sites shows that most bacteria are commensally acquired from the environment. Field mosquitoes were sampled in their breeding sites at the larval stage and maintained in their aquatic habitats until adult emergence. We propose that the acquisition of endobacteria occurred from the aquatic environment, and possibly by vertical transmission routes. Indeed transstadial transmission has been demonstrated in *Anopheles* mosquitoes [27], [51], and despite of “gut sterilization” during mosquito metamorphosis from pupae to adult, which is believed to contribute to a reduction of the larval microbiota [52], the bacterial clearance is not complete. Here, we show that the bacterial content of adult mosquitoes differed according to the breeding site where larvae were grown, and our results suggest that the composition of the midgut microbiota in adult mosquitoes relies on the bacterial richness of the native aquatic source.

The 454 sequencing allowed the identification of both commensal and symbiotic bacteria, giving a broad description of the mosquito midgut microbial community. Bacterial taxa, such as *Asaia* or *Burkholderia*, are known insect symbionts, contributing to beneficial associations and possibly to an enhanced pathogen resistance [35], [53]–[55]. We identified *Asaia spp.* as a predominant component of the gut microbiota in the mosquitoes from Nkolondom, representing more than 60% of sequence tags, but these bacteria also were found at lower abundance in all other mosquitoes even in those from the laboratory colony, which is indicative of a positive effect of this bacteria on mosquito fitness. Transmission of *Asaia* from adult to offspring occurs through an egg-mediated mechanism, but other modes of transmission, including contamination through the food source, have been described [35], [51], [56]. Several strains of *Asaia* colonize mosquito populations, including symbiotic and environmental isolates that follow distinct routes of transmission [35]. The difference of *Asaia* abundance in mosquitoes sampled in our two study sites, Nkolondom and Mvan, possibly underlies a genetic heterogeneity of the bacterium in the different environmental settings. By contrast to *Asaia*, *Burkholderia spp.* that were the dominant genera of the midgut microbiota in mosquitoes from Mvan, representing an average of 30% of sequence abundance, were not detected in the intestinal flora of the Ngousso colony. Thus, the infection by *Burkholderia* is not essential for growth and reproduction of the mosquito. Members of the genus *Burkholderia* are widespread in soil rhizospheres and plant surfaces, and some species are known to be associated with insects feeding on plants [41], [54], [57], [58]. In the latter case, the *Burkholderia* symbiont is environmentally acquired by the nymphs [54]. Studies on the association between *Burkholderia* and the insect midgut revealed mutualistic relationships, where the symbiont presence increases the insect fitness or protects the insect from entomopathogenic fungi [54], [55]. So far, as we know, the effect of the *Burkholderia* symbiont on malaria vectors is unknown. Further investigations on the microbiota dynamics through the mosquito life cycle, from egg to adult, are required to better define the nature of the microbe-insect associations and the most important microbial species critical for mosquito survival.

Despite a larger diversity of the gut microbiota in wild mosquitoes, most bacteria species are sparsely distributed between individual mosquitoes. Only 20 genera were found in more than 80% of individuals and 60 in >50%. In insects, the gut microbiota differs according to the food source, and in blood-sucking insects, bacterial content is higher after a blood meal [15], [23], [59], [60]. Here, because adult mosquitoes were fed the same diet, the high variability of taxa abundance results from individual variation, and the most abundant lineages represent the mosquito “core gut microbiota.” The existence of a core gut microbiota, by which different bacteria species are sharing metabolic functions and maintain the gut homeostasis, is now emerging [34], [61], [62]. Because alteration of the microbiota composition has been related to the development of diseases or health disorders, the next challenge is to define members of the microbial community and/or the metabolic interdependencies essential to preserve optimal gut homeostasis [34], [63].

The characterization of the mosquito core microbiota during the time course of *Plasmodium* infection will be a next step toward understanding the impact of gut bacteria on parasite development within the mosquito midgut. *P. falciparum* traverses the intestinal epithelium within 24 h after blood meal, at the peak of the digestion process; and whether parasites take advantage of intense competitive interactions for nutrient resources between bacteria to thwart the immune surveillance has to be investigated. Indeed, the gut microbiota is known to play an important role in protecting the host from potentially pathogenic microbes [64], [65]. Protection occurs through different processes: stimulation of the mosquito immune response, competition for binding sites or nutrients and production of toxins [65]–[67]. However, despite the beneficial role of the microbiota, pathogens, such as helminthes and viruses have developed strategies for exploiting the gut microbiota to promote their transmission [68], [69], [70]. For the mosquito vector, our understanding is still at an early stage for how the natural resident microbial flora of the mosquito midgut contributes to its resistance to the *Plasmodium* [15], [17], [18], [24].

In this study, we found that the abundance of *Enterobacteriaceae* is higher in *P. falciparum*-infected mosquitoes, suggesting that some microbe-parasite interactions may contribute to the successful development of the malaria parasite. However, whether *Enterobacteriaceae* have an effect on parasite survival or whether the increased level of *Enterobacteriaceae* is a consequence of *Plasmodium* development remains elusive. Alternatively, genetic factors, such as allelic polymorphism of immune genes, could regulate the variable levels of permissiveness of the mosquitoes as has been previously shown [71]. In contrast to our findings, previous studies reported the deleterious effect of bacterial infections on *Plasmodium* development in the mosquito [19], [23]–[25]. Of interest in this context, several *Enterobacteriaceae* strains were able to inhibit the development of *Plasmodium* species in the mosquito midgut, among them *Cedecea spp.*, *Serratia spp.*, and *Enterobacter spp.* isolated from *A. albimanus*, *A. stephensi*, or *A. arabiensis* [19], [22]–[24]. The Esp_Z *Enterobacter* strain isolated from *A. arabiensis* caught in Zambia [24] was not identified in any of the reads we analyzed. The possibility that this *Enterobacter* strain would have been absent from the PCR products because of competition with a different clade is unlikely as we used three different sets of primers. Therefore, we expect that in the gut of *A. gambiae* mosquitoes in Cameroon, the Esp_Z *Enterobacter* strain was below the 0.1% abundance threshold or absent. This *Enterobacter* strain was isolated in Zambia from wild-caught *A. arabiensis* mosquitoes, and differences in the mosquito species, as well as differences between the study areas, may explain why we did not find this bacterium in our material. Cirimotich *et al.* [24] recovered the Esp_Z on LB media, and culturing methods can lead to the artificial amplification of a bacterial strain present in minute amounts in an environmental sample. Therefore, it would be of interest to examine the presence/abundance of Esp_Z in wild-caught Zambian *A. arabiensis* using the methodologies described here. In our study, we analyzed the gut resident microbiota and revealed a positive correlation between commensal *Enterobacteriaceae* and *Plasmodium* infection, indicating that the *P. falciparum* infection phenotype under natural conditions results from more complex interactions than previously thought. Our data suggest a possible protective role of the *Enterobacteriaceae* on natural *P. falciparum* infection. Interestingly, it has been shown that commensal *Enterobacteriaceae* may promote intestinal homeostasis by enhancing immune receptors in the human colon [72]. For the mosquito, as described for the insect model *Drosophila* [73], gut homeostasis could be maintained through the renewal of the intestinal epithelial layer that can be altered upon bacterial killing or through immune regulation. A major challenge now will be to correlate our data with quantitative phenotyping of the immune system of the gut.

Previous reports on the susceptibility of the M and S molecular forms to *P. falciparum* infections relay contrasting findings [9], [10]. In Cameroon, mosquitoes of the two molecular forms collected in a sympatric area exhibited similar susceptibility to *P. falciparum* infection [10], whereas in Senegal, mosquitoes of the S form, derived from progenies of field-collected individuals, were more susceptible to *P. falciparum* than those of the M form [9]. In the present study with the mosquitoes collected in natural breeding sites and infected on the same blood donor, we found that the M form was more infected than the S form. However, a marked difference in the *P. falciparum* prevalence was observed according to the sampling site, and larger sample sizes of sympatric M and S populations of *A. gambiae* will be needed to further explore any difference of *Plasmodium* susceptibility between the two cryptic species. We propose that the composition of the gut microbiota may influence parasite transmission, which would explain the difference in infection levels between mosquito populations from diverse environments [9], [74].

The mosquito susceptibility to *Plasmodium* infection is under host genetic control, and several candidate genes have a recognized role in the establishment of the pathogen in the mosquito midgut [11]–[13]. However, how the mosquito gut microbiota influences *Plasmodium* transmission has to be unraveled. The mosquito gut ecosystem remains poorly understood, and elucidating the precise role of the symbiotic and commensal flora on the regulation of the insect immune response and on the infection course of pathogens, such as *Plasmodium* parasites, will be of great interest. Pathogens and microbes likely depend on similar mechanisms for interacting with their hosts, and a better knowledge of the mosquito-microbiota interactions would open new avenues for vector disease control through manipulation of gut microbial communities. Furthermore, unraveling these strategies mounted by the parasites to cooperate with the resident microbiota will allow a better understanding of co-evolution of host-pathogen interactions.

## Materials and Methods

### Ethics statement

All procedures involving human subjects used in this study were approved by the Cameroonian national ethical committee (statement 099/CNE/SE/09). The gametocyte carrier used in this study was enrolled as a volunteer after his parents had signed a written informed consent.

### Mosquito collection and sample characterization

*A. gambiae* mosquitoes were sampled in aquatic habitats at the L4 and pupae stages in four localities in Cameroon using standard dipping technique [75]. In each locality, breeding sites were inspected visually for presence of larval stages. At each breeding site, 10 dips were taken with a standard dipper (300 ml) and kept in a 5-liter container for transportation to the insectary at OCEAC. Anopheline larvae were identified morphologically; non-anopheline larvae and predators were removed. Larvae were kept in their original habitat water in a 3-liter plastic bucket and resulting pupae were collected daily for 2 days. Pupae were transferred to a plastic cup containing 20 ml of water from the breeding site, and the cup was placed in a 30×30 cm cage for emergence. The remaining larval collection was discarded after 2 days to avoid bias because of putative modifications of the biotic content of the aquatic habitats. Adult mosquitoes were maintained in standard insectary conditions (27±2°C, 85±5% RH, and 12 h light/dark) and provided with 8% sterile sucrose solution.

Female mosquitoes were fed on a single *P. falciparum* gametocyte carrier to avoid infection rate variability because of the blood donor. Infectious feeding was performed as previously described [71], [76]. Females, 3 to 5 days old, were starved for 24 h and allowed to feed on the *P. falciparum* gametocyte containing blood for 35 minutes through membrane feeders. Unfed and partially fed mosquitoes were removed by aspiration and discarded. Fully engorged females were kept in the insectary until dissections 8 days after the infectious blood meal. Mosquitoes were surface sterilized in 70% ethanol for 5 minutes, then rinsed twice in sterile PBS solution, and midguts were dissected and stored individually at −20°C until processing. DNA was extracted using the DNeasy Blood &Tissue Kit from Qiagen (Valencia, CA) and quantified (Nanodrop ND-1000, NanoDrop Technologies, Montchanin, DE, USA).

A 20-ng aliquot of DNAs was subjected to whole-genome amplification using the GenomiPhi V2 DNA Amplification Kit (GE HealthCare, Uppsala, Sweden), and the GenomiPhi templates served to characterize molecular forms of *A. gambiae* and the *P. falciparum* infection status. Molecular forms were determined according to Fanello *et al.* [77] and the identification of mosquitoes that successfully developed malaria infection using a *P. falciparum* specific PCR amplifying a Cox gene fragment [78].

### Sample selection for the metagenomic analysis

A total of 32 individual midguts were subjected to the 454-sequencing analysis. We included 2 samples of midguts dissected from mosquitoes of our local colony of *A. gambiae*, Ngousso. The Ngousso colony was established in January 2006 from larvae collected in breeding sites of an urbanized district of Yaounde, “Ngousso.” Larval collections were conducted during a 2-month period; mosquitoes were blood fed for oviposition and then PCR screened for molecular form of *A. gambiae*. Ngousso mosquitoes belong to the M molecular and Forest chromosomal forms. Since then, the colony has been routinely maintained at the OCEAC insectary. The Ngousso samples served to provide an overview of the bacterial content of laboratory mosquitoes reared under standard insectary conditions and as an experimental control in this study. The 30 remaining samples were chosen among field mosquitoes fed on blood from the same gametocyte carrier. We selected both *P. falciparum* positive and *P. falciparum* negative midguts to assess putative differences of microbiota between non-infected and infected individuals in our *P. falciparum*-challenged mosquitoes.

### 454 sequencing

For each individual midgut DNA sample, we generated three PCR amplicon libraries. We targeted 3 different hypervariable regions of the 16S ribosomal RNA to allow accurate detection of the bacterial communities in each sample. Indeed, previous analyses showed that the set of primers used for amplification can have a strong impact on the biodiversity revealed; some abundant clades in a given sample could be foreseen, depending on the primers used [45]. The S1 library targeting the V4 hypervariable region was obtained using the forward primer 535F (5′-GTGCCAGCAGCCGCGGTAATA-3′) and the reverse primer 789R (5′-GCGTGGACTACCAGGGTATCT-3′), the S2 library for the V5–6 region using the 326F (5′-CAAACAGGATTAGATACCCTG-3′) and the 1082R (5′-CGTTRCGGGACTTAACCCAACA-3′) primers, and the S3 library targeting the V5–6 region with the 1065F (5′-CAGGTGCTGCATGGCYGTCGT-3′) and the 1336R (5′-CGATTACTAGCGATTCC-3′) primers. Amplified DNA was purified and quantified using Picogreen fluorescent dye (Molecular Probes, Eurogen, OR). Individual libraries were processed for 454 sequencing by ligating the 454 adapters coupled to MID tags, allowing the multiplexing of samples. The amplicon libraries were pooled in two separate batches and sequenced. The MIDs and 454 linkers were ligated after the PCR amplification and the pyrotags were sequenced unidirectionally. Pyrosequencing was performed at Genoscreen (Lille, France) using a Genome Sequencer FLX Titanium (GS-FLX) system (Roche, Basel, Switzerland). In total, we recovered 663,651 sequence reads (tags) that were subjected to quality controls. All 454 sequences were deposited in Genebank (SRS281724.1 and SRS281725.1).

### Data processing and taxonomic assignment

Tags were extracted only if they contained the combination linker-MID-primer and the complement primer sequence at the 3′end. Tags were sorted in appropriate files according to their MID barcode and converted to the forward strand when necessary. A strict dereplication step was then applied that discarded tags with unidentified nucleotides (Ns) and those longer than 350 bp or shorter than 200 bp. Dereplicated tags were sorted by decreasing number of occurrences and clustered at k = 3 number of differences as described in Stoeck *et al.* [79]; this pipeline resulted in determining unique sequences. We next processed taxonomic assignments by implementing a new approach that clearly optimizes the successive assignments. We first extracted from the reference sequences of SILVA (release 106) domains corresponding to the various possible couples of the primers. Extraction was first performed requiring a perfect match between each primer and a sequence and, when no match was found, 1, 2 and 3 differences between each primer and a sequence were used successively. This pipeline then gave three reference databases, one per amplified 16S rDNA region, containing all reference amplicons putatively matching our tags. The S1, S2 and S3 tagged databases contained 424.634, 359.198 and 394.370 reference amplicons, respectively.

In a second step, all unique tags were assigned a taxon using a global alignment method. Each amplicon of the reference database was considered if it had at least 70% similarity with a tag. The list of reference amplicons was sorted by decreasing percentage of similarity and rounded to an integer. For taxonomic assignments, the reference sequence with the highest percentage was used, and taxonomy to a given level was obtained by the consensus of these taxonomies when more than one result emerged. For example, a tag with 98% similarity to the class *Gammaproteobacteria* and *Alphaproteobacteria* was only assigned to the phylum *Proteobacteria*. When similarity was <80%, sequences were not assigned. Tags were clustered into OTUs according to their consensus taxonomy. For each mosquito sample and each amplified 16S rDNA region, OTU abundances represent relative abundances, the number of reads for the given OTU divided by the total of tags in the SSU region of that sample.

Rarefaction curves were produced by plotting the number of unique sequence tags as a function of the number of randomly sampled tags. To generate rarefaction curves, we retained OTUs containing at least 2 sequence tags and encompassing the abundance threshold of 0.04% because rare sequences likely represent random sequencing errors and overestimate the overall diversity.

### Ecological indexes and statistical analysis

Ecological indexes such as richness and diversity indexes (Simpson, Shannon), were computed using the Vegan [80] and BiodiversityR [81] packages under the R software (available at http://www.R-project.org) [82]. Chao1, ACE1 and Jackknife richness estimators were calculated using the SPADE software [83]. Indexes were calculated using values from the genus taxonomic rank, the lowest rank obtained with the 454 technology.

Association between microbiota and environmental variables was assessed using a multivariate ordination test. We defined the different taxa present in the data set as “species variables” and the origin of the mosquitoes and the *P. falciparum* infection status as “environmental variables” for each individual. A detrended canonical correspondence analysis (DCA) was performed to determine the ordination method suitable for our data. The longest gradient we obtained was shorter than 3.0, indicating that the constrained form of linear ordination method, the RDA, was the most appropriate test [84]. Redundancy analysis was performed using Canoco v. 4.5 Software [85]. The environmental variables were set as dummy variables (0 or 1 values). RDA and associated Monte Carlo permutation tests (n = 499) were used to identify the measured environmental variable that contributed most significantly to the variation in the bacterial community data. The Monte Carlo test returns a *p* value associated with the effect of the environmental variable on the microbiota composition of the samples. Results were visualized on a biplot ordination diagram using CanoDraw extension.

## Supporting Information

Figure S1 **Rarefaction analyses for each mosquito midgut sample.** Saturation curves were generated by plotting the number of unique sequence tags as a function of the number of randomly sampled tags. Tags were clustered at k = 3 differences, and OTUs were set when containing at least 2 sequence tags and encompassing the abundance threshold of 0.04%.
(TIF)

Figure S2 **Redundancy analysis for gut bacterial communities (taxonomic rank = family) in field mosquitoes.** The length of arrows indicates the strength of correlation between the variable and the ordination scores. Blue arrow: bacterial classes, green arrow: environmental variables. The Monte Carlo permutation test was used to test the statistical significance of the relationship between environmental variables and the bacterial classes. “Family” and “Locality” variables segregate along the first axis, but “Entero” (*Enterobacteriaceae*) and “Infection” gathers along the second axis (*P*<0.05).
(TIF)

Table S1 **Molecular form identification and infection status of female** ***A. gambiae*** **mosquitoes collected at larval stage in different localities and challenged after emergence to a single** ***P. falciparum*** **donor.**
(DOC)

Table S2 **Mosquito characteristics and diversity indexes for each individual analyzed upon 454 sequencing of the S1 library.**
(DOC)

Table S3 **Main genera that composed the natural** ***A. gambiae*** **gut microbiota.**
(DOC)
