# Supplementary figures and images for: Midgut Microbiota of the Malaria Mosquito Vector Anopheles gambiae and Interactions with Plasmodium falciparum Infection
Source: PLoS Pathog. 2012 May 31;8(5):e1002742. doi: 10.1371/journal.ppat.1002742 (PMC3364955; doi:10.1371/journal.ppat.1002742)

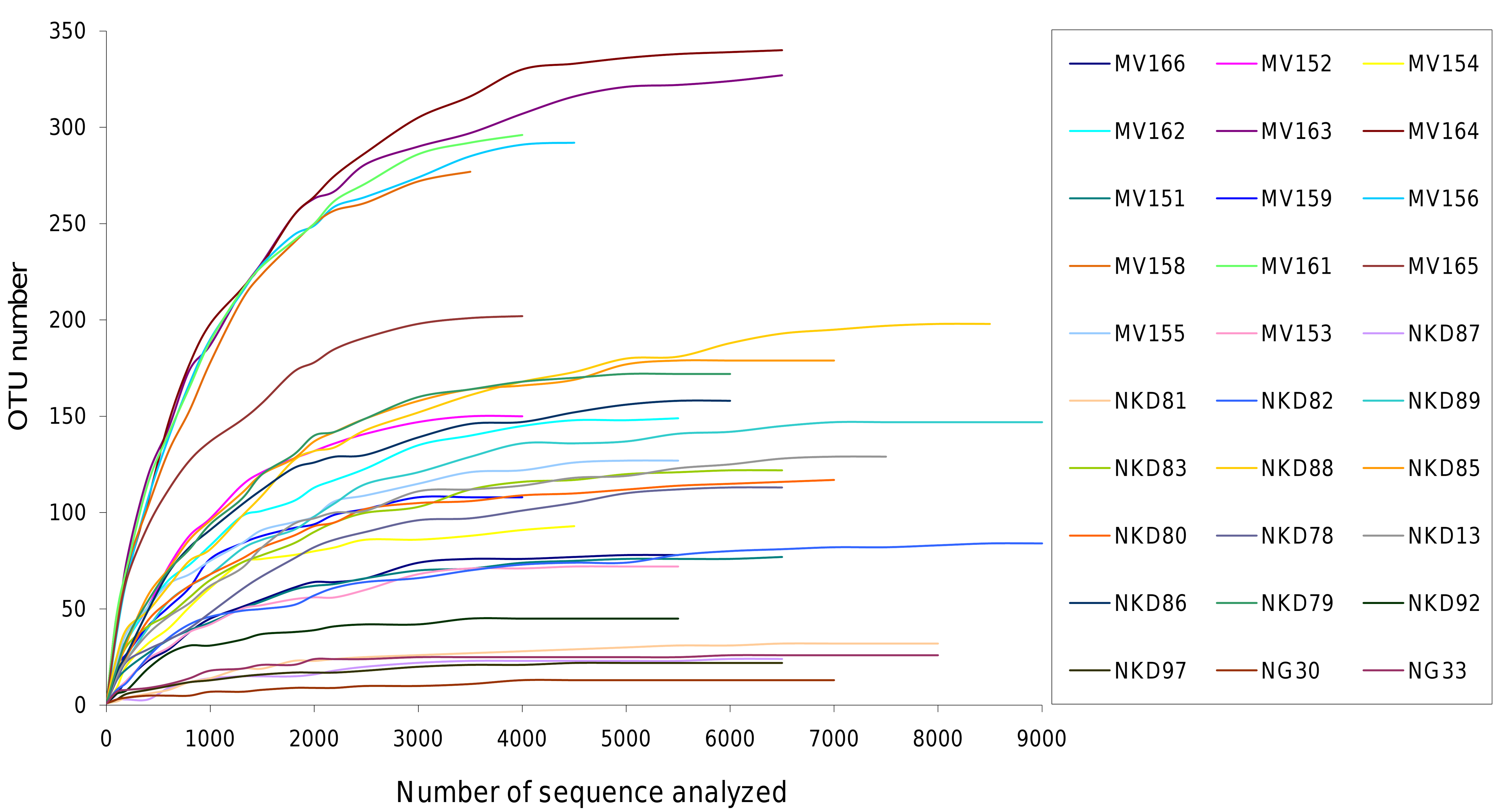

Supplement: Figure S1 — Rarefaction analyses for each mosquito midgut sample. Saturation curves were generated by plotting the number of unique sequence tags as a function of the number of randomly sampled tags. Tags were clustered at k = 3 differences, and OTUs were set when containing at least 2 sequence tags and encompassing the abundance threshold of 0.04%. (TIF) [file ppat.1002742.s001.tif]

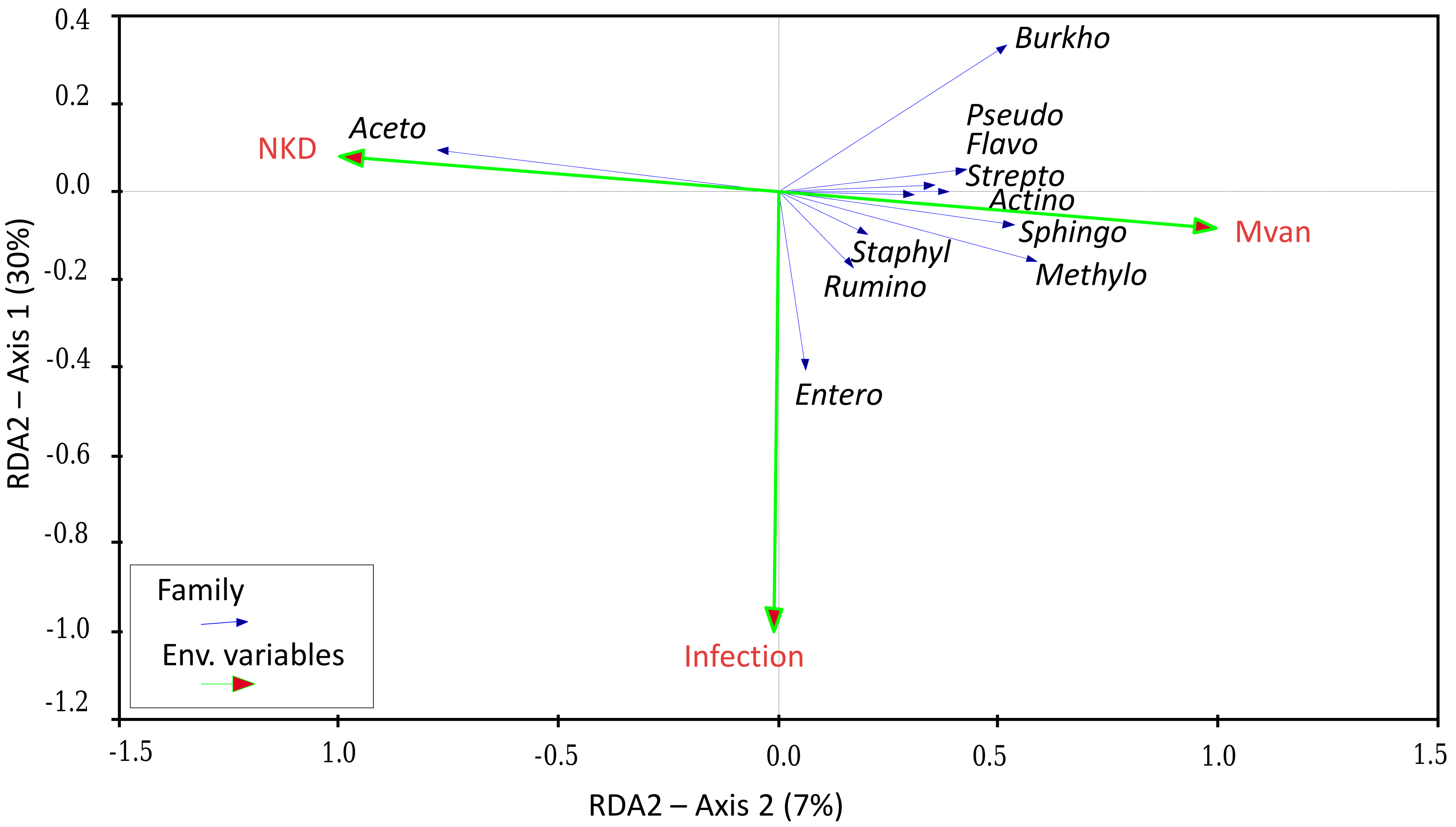

Supplement: Figure S2 — Redundancy analysis for gut bacterial communities (taxonomic rank = family) in field mosquitoes. The length of arrows indicates the strength of correlation between the variable and the ordination scores. Blue arrow: bacterial classes, green arrow: environmental variables. The Monte Carlo permutation test was used to test the statistical significance of the relationship between environmental variables and the bacterial classes. “Family” and “Locality” variables segregate along the first axis, but “Entero” (Enterobacteriaceae) and “Infection” gathers along the second axis (P<0.05). (TIF) [file ppat.1002742.s002.tif]
