# Supplementary material for: Midgut Microbiota of the Malaria Mosquito Vector Anopheles gambiae and Interactions with Plasmodium falciparum Infection
Source: PLoS Pathog. 2012 May 31;8(5):e1002742. doi: 10.1371/journal.ppat.1002742 (PMC3364955; doi:10.1371/journal.ppat.1002742)
Supplement: Table S1 — Molecular form identification and infection status of female A. gambiae mosquitoes collected at larval stage in different localities and challenged after emergence to a single P. falciparum donor. (DOC) [file ppat.1002742.s003.doc]

|  | **M form** | | **S form** | |
| --- | --- | --- | --- | --- |
| **Locality** | **Pf-** | **Pf+** | **Pf-** | **Pf+** |
| Mvan | 8 | 11 | 0 | 0 |
| Nkolbisson | 6 | 5 | 5 | 2 |
| Nkolkumu | 3 | 0 | 17 | 2 |
| Nkolondom | 0 | 0 | 21 | 12 |

Pf-, uninfected mosquitoes; Pf+, infected mosquitoes. The infection status was determined by presence/absence of oocysts in the midgut at day 8 post-challenge.
