# Supplementary material for: Midgut Microbiota of the Malaria Mosquito Vector Anopheles gambiae and Interactions with Plasmodium falciparum Infection
Source: PLoS Pathog. 2012 May 31;8(5):e1002742. doi: 10.1371/journal.ppat.1002742 (PMC3364955; doi:10.1371/journal.ppat.1002742)
Supplement: Table S2 — Mosquito characteristics and diversity indexes for each individual analyzed upon 454 sequencing of the S1 library. (DOC) [file ppat.1002742.s004.doc]

| **Gut ID** | **Locality** | **M/S form** | ***Pf* status** | **Richness** | **Chao1** | **ACE1** | **Jackknife** | **Simpson** | **Shannon** |
| --- | --- | --- | --- | --- | --- | --- | --- | --- | --- |
| NKD78 | NKD | S | + | 82 | 93.6 | 99.4 | 100.0 | 0.344 | 1.058 |
| NKD79 | NKD | S | + | 81 | 105.0 | 88.6 | 93.0 | 0.621 | 1.934 |
| NKD80 | NKD | S | + | 87 | 115.0 | 118.4 | 113.0 | 0.619 | 1.469 |
| NKD86 | NKD | S | + | 94 | 116.0 | 113.0 | 116.0 | 0.546 | 1.639 |
| NKD92 | NKD | S | + | 30 | 50.2 | 42.1 | 41.0 | 0.352 | 0.653 |
| NKD97 | NKD | S | + | 10 | - | 13.0 | 14.7 | 0.060 | 0.177 |
| NKD03 | NKD | S | + | 92 | 98.5 | 102.5 | 106.0 | 0.360 | 1.175 |
| NKD81 | NKD | S | - | 36 | 91.1 | 197.1 | 57.0 | 0.076 | 0.234 |
| NKD82 | NKD | S | - | 69 | 129.1 | 139.4 | 98.0 | 0.426 | 0.903 |
| NKD83 | NKD | S | - | 88 | 99.1 | 104.4 | 108.0 | 0.573 | 1.429 |
| NKD85 | NKD | S | - | 102 | 113.6 | 112.0 | 118.0 | 0.596 | 1.810 |
| NKD87 | NKD | S | - | 21 | 45.5 | 27.8 | 28.0 | 0.021 | 0.087 |
| NKD88 | NKD | S | - | 94 | 98.5 | 98.4 | 103.0 | 0.661 | 1.936 |
| NKD89 | NKD | S | - | 101 | 129.1 | 143.7 | 131.0 | 0.605 | 1.555 |
| MV151 | Mvan | M | + | 58 | 80.2 | 83.9 | 78.0 | 0.475 | 1.120 |
| MV153 | Mvan | M | + | 37 | 41.9 | 43.3 | 44.0 | 0.575 | 1.146 |
| MV155 | Mvan | M | + | 85 | 107.2 | 121.1 | 109.0 | 0.611 | 1.561 |
| MV156 | Mvan | M | + | 115 | 129.4 | 126.7 | 132.0 | 0.915 | 3.105 |
| MV158 | Mvan | M | + | 102 | 129.0 | 115.5 | 120.0 | 0.917 | 3.105 |
| MV159 | Mvan | M | + | 72 | 112.9 | 155.6 | 102.0 | 0.470 | 1.260 |
| MV161 | Mvan | M | + | 94 | 102.2 | 103.7 | 108.0 | 0.867 | 2.774 |
| MV165 | Mvan | M | + | 80 | 84.0 | 84.2 | 88.0 | 0.833 | 2.669 |
| MV152 | Mvan | M | - | 82 | 108.0 | 117.9 | 108.0 | 0.500 | 1.544 |
| MV154 | Mvan | M | - | 50 | 69.6 | 68.7 | 64.0 | 0.486 | 1.189 |
| MV162 | Mvan | M | - | 82 | 119.8 | 110.8 | 105.0 | 0.570 | 1.611 |
| MV163 | Mvan | M | - | 113 | 131.3 | 126.6 | 129.0 | 0.917 | 3.192 |
| MV164 | Mvan | M | - | 118 | 138.6 | 134.4 | 135.0 | 0.908 | 3.087 |
| MV166 | Mvan | M | - | 61 | 87.5 | 107.3 | 84.0 | 0.193 | 0.641 |
| NG30 | labo | M | nc | 9 | 10.0 | 12.1 | 11.0 | 0.045 | 0.132 |
| NG33 | labo | M | nc | 23 | 43.3 | 45.3 | 32.0 | 0.164 | 0.434 |

NKD, Nkolondom; labo, laboratory-reared mosquitoes; nc, non-challenged.mosquitoes. The *P. falciparum* infection status (Pf status) was determined by presence (+) or absence (-) of oocysts in the midgut at day 8 post-challenge. Chao1, ACE1 and Jackknife richness estimators were calculated using SPADE software. Richness and diversity indexes (Simpson, Shannon) were computed using the Vegan and BiodiversityR packages under the R software.
