# Supplementary material for: Midgut Microbiota of the Malaria Mosquito Vector Anopheles gambiae and Interactions with Plasmodium falciparum Infection
Source: PLoS Pathog. 2012 May 31;8(5):e1002742. doi: 10.1371/journal.ppat.1002742 (PMC3364955; doi:10.1371/journal.ppat.1002742)
Supplement: Table S3 — Main genera that composed the natural A. gambiae gut microbiota. (DOC) [file ppat.1002742.s005.doc]

| *Genus* | N | Microbiota percentage* | Ecological characteristics |
| --- | --- | --- | --- |
| *Asaia* | 28 | 38.76 [26.33-51.18] | Flowers |
| *Burkholderia* | 27 | 19.37 [9.52-29.23] | Ubiquitous |
| *Acinetobacter* | 27 | 3.82 [2.15-5.49] | Ubiquitous |
| *Ralstonia* | 27 | 2.96 [1.73-4.20] | Soil, plants |
| *Methylobacterium* | 27 | 1.65 [0.86-2.43] | Soil, plants |
| *Sphingomonas* | 26 | 2.47 [1.44-3.49] | Water, soil, flowers |
| *Pseudomonas* | 26 | 1.65 [1.07-2.23] | Water, flowers |
| *Stenotrophomonas* | 25 | 0.39 [0.23-0.55] | Soil, water |
| *Streptococcus* | 24 | 0.42 [0.25-0.59] | Ubiquitous |
| *Comamonas* | 24 | 0.30 [0.17-0.43] | Soil, water |
| *Sediminibacterium* | 24 | 0.25 [0.16-0.35] | Soil |
| *Escherichia-Shigella* | 24 | 0.18 [0.07-0.29] | Commensal |
| *Bradyrhizobium* | 23 | 0.20 [0.09-0.30] | Soil |
| *Staphylococcus* | 22 | 0.23 [0.13-0.32] | Ubiquitous |
| *Fusobacterium* | 20 | 0.11 [0.04-0.18] | Commensal |
| *Elizabethkingia* | 19 | 0.10 [0.04-0.16] | Soil, water |
| *Gluconacetobacter* | 18 | 0.27 [0.09-0.44] | Flowers |
| *Neisseria* | 16 | 0.13 [0.05-0.21] | Commensal |
| *Schlegelella* | 15 | 0.14 [0.02-0.27] | Soil, water |
| *Prevotella* | 15 | 0.12 [0.01-0.22] | Commensal |
| *Cedecea* | 13 | 1.00 [0.00-2.31] | Commensal |
| *Serratia* | 12 | 4.20 [0.00-10.95] | Water, soil, flowers |

N, number of mosquitoes containing the genus; *, average percentage (% and 95%IC) of the genus in total microbiota over the 28 field mosquitoes. “Ecological characteristics” indicate the main biotopes where the genus has been previously described.
